# Multi‐ancestry genome‐wide gene × age interaction study identifies novel loci associated with late‐onset Alzheimer’s Disease

**DOI:** 10.1002/alz.093279

**Published:** 2025-01-03

**Authors:** Tong Tong, Congcong Zhu, John J. Farrell, Eden R. Martin, Margaret A Pericak‐Vance, Li‐San Wang, Gerard D. Schellenberg, William S. Bush, Jonathan L. Haines, Kathryn L. Lunetta, Xiaoling Zhang, Lindsay A. Farrer

**Affiliations:** ^1^ Boston University Bioinformatics Program, Boston, MA USA; ^2^ Department of Medicine (Biomedical Genetics), Boston University Chobanian & Avedisian School of Medicine, Boston, MA USA; ^3^ John P. Hussman Institute for Human Genomics, University of Miami Miller School of Medicine, Miami, FL USA; ^4^ Department of Pathology and Laboratory Medicine, University of Pennsylvania Perelman School of Medicine, Philadelphia, PA USA; ^5^ Department of Population and Quantitative Health Sciences, Case Western Reserve University, Cleveland, OH USA; ^6^ Department of Biostatistics, Boston University School of Public Health, Boston, MA USA; ^7^ Department of Ophthalmology, Boston University Chobanian & Avedisian School of Medicine, Boston, MA USA; ^8^ Department of Neurology, Boston University Chobanian & Avedisian School of Medicine, Boston, MA USA; ^9^ Department of Epidemiology, Boston University School of Public Health, Boston, MA USA

## Abstract

**Background:**

Age is the largest risk factor for late‐onset Alzheimer’s Disease (LOAD). Although >80 genetic loci have been associated with LOAD, little is known about the age dependencies of these associations except the *APOE* region.

**Method:**

We performed cross‐ancestry and ancestry‐specific genome‐wide gene‐age interaction and age‐stratified association study using TOPMed‐imputed genome‐wide association study (GWAS) data from Alzheimer’s Disease Genetics Consortium (ADGC) including 34,833 non‐Hispanic Whites (NHW), 7,264 African Americans (AA), 3,232 East Asians (EA), and 2,024 Caribbean Hispanics (CH) aged 60 years and older. We chose an approximate median age‐at‐onset threshold of 75 to dichotomize participants into younger (N_case_:N_control_ = 10, 905:11,624) and older (N_case_:N_control_ = 9,982:14,842) groups. We first evaluated the association of LOAD with the interaction of SNP × age within each cohort using a linear mixed‐effect model including a binary term for the age group, numeric age, sex, and the first 5 principal components (PCs) of ancestry. Results from each cohort were combined using a fixed‐effect model to jointly estimate the regression coefficients of SNP and SNP × age terms, both within‐ancestry and cross‐ancestry analyses. We also performed an age‐stratified analysis that included all controls regardless of age to increase power and using the same linear model without the interaction term. Results across cohorts were combined by meta‐analysis using a fixed‐effect model within‐ancestry and a modified random‐effect model across ancestry.

**Result:**

In cross‐ancestry joint meta‐analysis, we identified 6 genome‐wide significant (GWS) loci, 4 of which showed nominal evidence of age‐dependent association with LOAD, including *CD2AP* (rs5876027, P_Joint_ = 2.33 × 10^‐8^, P_SNP×age_ = 0.043), *PICALM* (rs583162, P_Joint_ = 2.34 × 10^‐8^, P_SNP×age_ = 7.60 × 10^‐3^), *APOE* (rs429358, P_Joint_ = 0, P_SNP×age_ = 1.58 × 10^‐7^) and *LILRA5* (rs1761461, P_Joint_ = 4.86 × 10^‐8^, P_SNP×age_ = 0.032), for the first time. Within‐ancestry joint meta‐analysis identified novel associations with *VXN* (rs146432976, P_Joint_ = 2.29 × 10^‐8^, P_SNP×age_ = 3.08 × 10^‐5^) in AAs and *PALM2‐AKAP2* (rs67191673, P_Joint_ = 3.46 × 10^‐9^, P_SNP×age_ = 0.013) in EAs. *CR1* (rs6661489, P_Joint_ = 3.54 × 10^‐8^) and *TREM2* (rs75932628, P_Joint_ = 8.61 × 10^‐10^) were associated with LOAD in NHWs but showed no evidence of age‐dependent effects. In cross‐ancestry age‐stratified GWAS, *BIN1*, *PICALM*, and *APOE* region genes reached GWS in both strata, while *MS4A6A* was only significant in older group (rs7232, OR = 0.88, P = 4.23 × 10^‐8^). A GWS association with *PALM2‐AKAP2* (rs67191673, OR = 1.94, P = 3.64 × 10^‐9^) was identified in the EA younger group.

**Conclusion:**

We identified several ancestry‐specific age‐dependent association with previously established and novel loci.

